# Supplementary material for: Deletion of wfs1 Impairs Oligodendrocyte Precursor Cells Dorsal Distribution and Myelination Through the wfs1-hmgcs1 Axis in Zebrafish
Source: Biology (Basel). 2026 Aug 16;15(16):1402. doi: 10.3390/biology15161402 (PMC13510151; doi:10.3390/biology15161402)
Supplement: Supplementary file 1 [file biology-15-01402-s001.zip › File S1-Transcriptome sequencing data and differentially expressed genes (DEGs) in wfs1-- zebrafish at 5 dpf/2.KEGG/wfs1KOvsWT/GSEA/wfs1KO/FOXO_SIGNALING_PATHWAY/FOXO_SIGNALING_PATHWAY(DRE04068).html]

Details for gene set FOXO\_SIGNALING\_PATHWAY(DRE04068)[GSEA]

|  || Dataset | deg.sample |
| Phenotype | sample.cls |
| Upregulated in class | wfs1KO |
| GeneSet | FOXO\_SIGNALING\_PATHWAY(DRE04068) |
| Enrichment Score (ES) | 0.23812588 |
| Normalized Enrichment Score (NES) | 1.240001 |
| Nominal p-value | 0.1537037 |
| FDR q-value | 0.32069385 |
| FWER p-Value | 1.0 |
Table: GSEA Results Summary

  

Fig 1: Enrichment plot: FOXO\_SIGNALING\_PATHWAY(DRE04068)      
 Profile of the Running ES Score & Positions of GeneSet Members on the Rank Ordered List

  

| PROBE | DESCRIPTION (from dataset) | GENE SYMBOL | GENE\_TITLE | RANK IN GENE LIST | RANK METRIC SCORE | RUNNING ES | CORE ENRICHMENT || 1 | 324140\_1 | sgk1 |  |  | 109 | 4.683 | 0.0321 | Yes |
| 2 | 406473 | pck2 |  |  | 487 | 3.031 | 0.0432 | Yes |
| 3 | 566841 | crebbpa |  |  | 530 | 2.904 | 0.0639 | Yes |
| 4 | 431763 | gadd45ab |  |  | 713 | 2.560 | 0.0776 | Yes |
| 5 | 100000252\_3 | pik3r3b |  |  | 716 | 2.550 | 0.0968 | Yes |
| 6 | 562552 | zgc:171775 |  |  | 839 | 2.330 | 0.1107 | Yes |
| 7 | 117508 | klf2a |  |  | 888 | 2.251 | 0.1262 | Yes |
| 8 | 117508\_1 | klf2a |  |  | 938 | 2.182 | 0.1412 | Yes |
| 9 | 767751\_1 | ccnb3 |  |  | 975 | 2.119 | 0.1562 | Yes |
| 10 | 559723 | tgfb2l |  |  | 1105 | 1.990 | 0.1672 | Yes |
| 11 | 245702 | igf1rb |  |  | 1196 | 1.904 | 0.1788 | Yes |
| 12 | 565576 | plk2b |  |  | 1324 | 1.789 | 0.1883 | Yes |
| 13 | 280649\_1 | plk1 |  |  | 1335 | 1.777 | 0.2015 | Yes |
| 14 | 326974\_1 | gabarapa |  |  | 1391 | 1.732 | 0.2129 | Yes |
| 15 | 100151416 | cdkn1a |  |  | 1627 | 1.584 | 0.2175 | Yes |
| 16 | 100000252 | pik3r3b |  |  | 1965 | 1.382 | 0.2174 | Yes |
| 17 | 563560 | ikbkb |  |  | 2417 | 1.202 | 0.2122 | Yes |
| 18 | 393548 | gadd45aa |  |  | 2430 | 1.197 | 0.2209 | Yes |
| 19 | 565038 | ccnd2b |  |  | 3069 | 1.021 | 0.2086 | Yes |
| 20 | 100000242\_1 | zgc:92606 |  |  | 3071 | 1.020 | 0.2163 | Yes |
| 21 | 497646 | gadd45bb |  |  | 3089 | 1.017 | 0.2234 | Yes |
| 22 | 64617\_2 | s1pr1 |  |  | 3164 | 1.000 | 0.2287 | Yes |
| 23 | 790919 | g6pc3 |  |  | 3212 | 0.991 | 0.2347 | Yes |
| 24 | 266794 | ccng2 |  |  | 3422 | 0.947 | 0.2353 | Yes |
| 25 | 572001 | irs2b |  |  | 3794 | 0.869 | 0.2302 | Yes |
| 26 | 436769 | homer1b |  |  | 3989 | 0.833 | 0.2304 | Yes |
| 27 | 404211 | pik3r2 |  |  | 4059 | 0.817 | 0.2344 | Yes |
| 28 | 30068 | cat |  |  | 4462 | 0.750 | 0.2274 | Yes |
| 29 | 336116 | bnip3 |  |  | 4475 | 0.748 | 0.2327 | Yes |
| 30 | 572074 | prkaa2 |  |  | 4710 | 0.717 | 0.2308 | Yes |
| 31 | 30637\_1 | mdm2 |  |  | 4722 | 0.715 | 0.2358 | Yes |
| 32 | 768159 | si:ch211-195b13.1 |  |  | 4819 | 0.702 | 0.2381 | Yes |
| 33 | 100001198 | prkaa1 |  |  | 5040 | 0.675 | 0.2363 | No |
| 34 | 100006858 | csnk1e |  |  | 5879 | 0.599 | 0.2144 | No |
| 35 | 735295 | faslg |  |  | 6149 | 0.572 | 0.2103 | No |
| 36 | 100002318 | mapk13 |  |  | 6155 | 0.572 | 0.2144 | No |
| 37 | 768121 | foxo1a |  |  | 6230 | 0.565 | 0.2164 | No |
| 38 | 58092\_1 | smad3a |  |  | 6307 | 0.555 | 0.2182 | No |
| 39 | 406304 | gadd45ba |  |  | 6674 | 0.522 | 0.2106 | No |
| 40 | 559273\_1 | ep300a |  |  | 6707 | 0.519 | 0.2135 | No |
| 41 | 368390 | plk4 |  |  | 6831 | 0.507 | 0.2135 | No |
| 42 | 402991 | gadd45ga |  |  | 6915 | 0.499 | 0.2147 | No |
| 43 | 334202 | plk3 |  |  | 6917 | 0.499 | 0.2184 | No |
| 44 | 65236 | mapk8b |  |  | 6998 | 0.492 | 0.2196 | No |
| 45 | 567969 | foxo1b |  |  | 7019 | 0.490 | 0.2227 | No |
| 46 | 561737 | pik3ca |  |  | 7195 | 0.474 | 0.2208 | No |
| 47 | 100151181 | si:dkey-239n17.4 |  |  | 7340 | 0.463 | 0.2197 | No |
| 48 | 405850 | foxg1b |  |  | 7385 | 0.461 | 0.2219 | No |
| 49 | 562552\_1 | zgc:171775 |  |  | 7885 | 0.432 | 0.2094 | No |
| 50 | 100001936 | bcl6ab |  |  | 8537 | 0.400 | 0.1919 | No |
| 51 | 245701\_1 | igf1ra |  |  | 8653 | 0.391 | 0.1912 | No |
| 52 | 393707\_3 | bcl6a |  |  | 8656 | 0.391 | 0.1941 | No |
| 53 | 368316 | ccnb2 |  |  | 8799 | 0.381 | 0.1925 | No |
| 54 | 768202 | pdpk1a |  |  | 9086 | 0.361 | 0.1862 | No |
| 55 | 402792 | zgc:73226 |  |  | 9206 | 0.353 | 0.1851 | No |
| 56 | 378972 | akt2 |  |  | 9293 | 0.347 | 0.1851 | No |
| 57 | 393891 | fbxo32 |  |  | 9304 | 0.346 | 0.1874 | No |
| 58 | 553319 | prkag3a |  |  | 9526 | 0.332 | 0.1829 | No |
| 59 | 30663 | rag1 |  |  | 9722 | 0.320 | 0.1792 | No |
| 60 | 563884 | tgfb1b |  |  | 9725 | 0.320 | 0.1815 | No |
| 61 | 406507 | gadd45gb.1 |  |  | 9742 | 0.319 | 0.1835 | No |
| 62 | 562720 | mapk8a |  |  | 9888 | 0.310 | 0.1812 | No |
| 63 | 793200 | gabarapb |  |  | 9960 | 0.306 | 0.1813 | No |
| 64 | 560317 | si:dkey-222b8.1 |  |  | 10062 | 0.300 | 0.1804 | No |
| 65 | 65237\_1 | mapk14a |  |  | 10301 | 0.285 | 0.1750 | No |
| 66 | 570761 | prkag2a |  |  | 10629 | 0.265 | 0.1667 | No |
| 67 | 393228\_1 | zgc:55558 |  |  | 10754 | 0.257 | 0.1648 | No |
| 68 | 563181 | map2k2a |  |  | 10792 | 0.256 | 0.1655 | No |
| 69 | 369194 | tgfb2 |  |  | 10813 | 0.254 | 0.1668 | No |
| 70 | 245701 | igf1ra |  |  | 11103 | 0.239 | 0.1595 | No |
| 71 | 403037 | fbxo25 |  |  | 11189 | 0.234 | 0.1586 | No |
| 72 | 570965\_1 | atg12 |  |  | 11314 | 0.226 | 0.1564 | No |
| 73 | 560578 | tgfbr1a |  |  | 11347 | 0.225 | 0.1571 | No |
| 74 | 326283 | smad3b |  |  | 11759 | 0.203 | 0.1457 | No |
| 75 | 100000750 | g6pcb |  |  | 11811 | 0.200 | 0.1456 | No |
| 76 | 368415 | ptenb |  |  | 11887 | 0.196 | 0.1447 | No |
| 77 | 245700 | insrb |  |  | 12333 | 0.172 | 0.1320 | No |
| 78 | 100134973 | il7r |  |  | 12411 | 0.169 | 0.1308 | No |
| 79 | 368329 | cdkn1bb |  |  | 12428 | 0.168 | 0.1316 | No |
| 80 | 794669 | gb:cr929477 |  |  | 12490 | 0.165 | 0.1309 | No |
| 81 | 402862 | cdkn1ba |  |  | 12928 | 0.144 | 0.1182 | No |
| 82 | 570956 | sgk2a |  |  | 13200 | 0.130 | 0.1106 | No |
| 83 | 406715 | cdk2 |  |  | 13336 | 0.124 | 0.1073 | No |
| 84 | 567111 | crebbpb |  |  | 13470 | 0.117 | 0.1040 | No |
| 85 | 30274 | foxg1a |  |  | 13634 | 0.109 | 0.0997 | No |
| 86 | 100329897 | irs4a |  |  | 13886 | 0.097 | 0.0925 | No |
| 87 | 30681 | mapk12a |  |  | 13899 | 0.097 | 0.0929 | No |
| 88 | 569698 | mapk10 |  |  | 13907 | 0.096 | 0.0934 | No |
| 89 | 561670 | sos2 |  |  | 14111 | 0.087 | 0.0876 | No |
| 90 | 100149794 | akt3b |  |  | 14249 | 0.080 | 0.0839 | No |
| 91 | 794462 | LOC794462 |  |  | 14402 | 0.074 | 0.0797 | No |
| 92 | 245699 | insra |  |  | 14497 | 0.069 | 0.0772 | No |
| 93 | 58025 | ccnb1 |  |  | 14783 | 0.056 | 0.0687 | No |
| 94 | 266756 | mapk14b |  |  | 14849 | 0.053 | 0.0670 | No |
| 95 | 794088\_1 | ptena |  |  | 15035 | 0.044 | 0.0615 | No |
| 96 | 559281 | irs1 |  |  | 15119 | 0.041 | 0.0592 | No |
| 97 | 568155\_1 | bnip4 |  |  | 15432 | 0.025 | 0.0495 | No |
| 98 | 30296 | foxo3b |  |  | 15498 | 0.022 | 0.0477 | No |
| 99 | 403036 | gabarapl2 |  |  | 15513 | 0.022 | 0.0474 | No |
| 100 | 565612 | ep300b |  |  | 15569 | 0.019 | 0.0458 | No |
| 101 | 30380 | nras |  |  | 15635 | 0.015 | 0.0439 | No |
| 102 | 799608 | ccnd2a |  |  | 15743 | 0.009 | 0.0406 | No |
| 103 | 406308 | grb2b |  |  | 15876 | 0.003 | 0.0364 | No |
| 104 | 30767 | stat3 |  |  | 16064 | -0.004 | 0.0305 | No |
| 105 | 100885851 | il6 |  |  | 16069 | -0.004 | 0.0304 | No |
| 106 | 791141 | zgc:158659 |  |  | 16140 | -0.006 | 0.0283 | No |
| 107 | 378478 | egfra |  |  | 16429 | -0.015 | 0.0193 | No |
| 108 | 559111 | smad4a |  |  | 16436 | -0.016 | 0.0192 | No |
| 109 | 335232 | prkab1b |  |  | 16449 | -0.016 | 0.0190 | No |
| 110 | 563460 | nlk2 |  |  | 16472 | -0.017 | 0.0184 | No |
| 111 | 563180 | g6pca.2 |  |  | 16729 | -0.023 | 0.0105 | No |
| 112 | 559273 | ep300a |  |  | 16774 | -0.025 | 0.0093 | No |
| 113 | 553758 | usp7 |  |  | 17117 | -0.037 | -0.0012 | No |
| 114 | 560446 | si:dkey-101k6.5 |  |  | 17193 | -0.040 | -0.0033 | No |
| 115 | 563719 | foxo4 |  |  | 17382 | -0.049 | -0.0088 | No |
| 116 | 436905 | prkab1a |  |  | 17733 | -0.064 | -0.0194 | No |
| 117 | 100149273 | mapk9 |  |  | 17865 | -0.070 | -0.0230 | No |
| 118 | 405892 | nlk1 |  |  | 17881 | -0.071 | -0.0229 | No |
| 119 | 394174 | pik3cd |  |  | 17974 | -0.074 | -0.0253 | No |
| 120 | 100148413 | plk2a |  |  | 18172 | -0.084 | -0.0309 | No |
| 121 | 550537 | stk11 |  |  | 18181 | -0.084 | -0.0305 | No |
| 122 | 404211\_1 | pik3r2 |  |  | 18555 | -0.101 | -0.0415 | No |
| 123 | 415185 | mapk11 |  |  | 18679 | -0.107 | -0.0445 | No |
| 124 | 564523 | sgk3 |  |  | 18781 | -0.112 | -0.0469 | No |
| 125 | 30739 | tgfbr2b |  |  | 18789 | -0.113 | -0.0463 | No |
| 126 | 117509 | klf2b |  |  | 18948 | -0.120 | -0.0503 | No |
| 127 | 559273\_2 | ep300a |  |  | 19072 | -0.125 | -0.0533 | No |
| 128 | 378727 | pck1 |  |  | 19075 | -0.125 | -0.0524 | No |
| 129 | 678619 | prkag3b |  |  | 19261 | -0.133 | -0.0572 | No |
| 130 | 560549 | akt3a |  |  | 19324 | -0.136 | -0.0581 | No |
| 131 | 399480 | mapk3 |  |  | 19514 | -0.144 | -0.0630 | No |
| 132 | 406728 | map2k1 |  |  | 19540 | -0.145 | -0.0627 | No |
| 133 | 100170795 | prkab2 |  |  | 19617 | -0.149 | -0.0640 | No |
| 134 | 369195 | tgfb3 |  |  | 19890 | -0.161 | -0.0713 | No |
| 135 | 245701\_2 | igf1ra |  |  | 20190 | -0.174 | -0.0794 | No |
| 136 | 557109 | raf1b |  |  | 20203 | -0.175 | -0.0785 | No |
| 137 | 100142648 | foxg1d |  |  | 20244 | -0.177 | -0.0784 | No |
| 138 | 393285 | irs2a |  |  | 20347 | -0.181 | -0.0803 | No |
| 139 | 561541 | si:dkey-11f5.5 |  |  | 20574 | -0.192 | -0.0859 | No |
| 140 | 792928 | tgfbr1b |  |  | 20664 | -0.197 | -0.0873 | No |
| 141 | 567111\_1 | crebbpb |  |  | 20683 | -0.198 | -0.0863 | No |
| 142 | 493609\_1 | grb2a |  |  | 20689 | -0.198 | -0.0850 | No |
| 143 | 393707\_1 | bcl6a |  |  | 20756 | -0.201 | -0.0855 | No |
| 144 | 568316 | prkag2b |  |  | 21087 | -0.220 | -0.0943 | No |
| 145 | 494532 | foxo3a |  |  | 21163 | -0.224 | -0.0949 | No |
| 146 | 359834 | tgfb1a |  |  | 21423 | -0.237 | -0.1013 | No |
| 147 | 30222 | ccnd1 |  |  | 21522 | -0.242 | -0.1026 | No |
| 148 | 436866 | tnfsf10 |  |  | 21725 | -0.253 | -0.1070 | No |
| 149 | 559050 | sgk2b |  |  | 22037 | -0.269 | -0.1148 | No |
| 150 | 245701\_3 | igf1ra |  |  | 22143 | -0.275 | -0.1160 | No |
| 151 | 403064 | atm |  |  | 22311 | -0.285 | -0.1191 | No |
| 152 | 566735 | insb |  |  | 22312 | -0.285 | -0.1170 | No |
| 153 | 58092 | smad3a |  |  | 22399 | -0.290 | -0.1175 | No |
| 154 | 100190951 | bcl2l11 |  |  | 22410 | -0.290 | -0.1156 | No |
| 155 | 403065 | braf |  |  | 22710 | -0.308 | -0.1227 | No |
| 156 | 559723\_1 | tgfb2l |  |  | 22957 | -0.324 | -0.1280 | No |
| 157 | 555576 | grm1a |  |  | 23212 | -0.342 | -0.1335 | No |
| 158 | 360144\_1 | mapk1 |  |  | 23446 | -0.357 | -0.1381 | No |
| 159 | 100002263 | pik3cb |  |  | 23508 | -0.361 | -0.1373 | No |
| 160 | 335799 | sod2 |  |  | 23556 | -0.364 | -0.1360 | No |
| 161 | 557176 | pik3r1 |  |  | 23610 | -0.367 | -0.1349 | No |
| 162 | 65237 | mapk14a |  |  | 23905 | -0.386 | -0.1412 | No |
| 163 | 794669\_1 | gb:cr929477 |  |  | 24180 | -0.405 | -0.1468 | No |
| 164 | 100150246\_1 | grm1b |  |  | 24426 | -0.416 | -0.1514 | No |
| 165 | 100002225 | map2k2b |  |  | 24736 | -0.433 | -0.1579 | No |
| 166 | 393287 | chuk |  |  | 25038 | -0.449 | -0.1640 | No |
| 167 | 797132 | sirt1 |  |  | 25130 | -0.456 | -0.1634 | No |
| 168 | 100534937 | LOC100534937 |  |  | 25301 | -0.467 | -0.1652 | No |
| 169 | 100331892 | rbl2 |  |  | 25425 | -0.478 | -0.1655 | No |
| 170 | 557810 | mapk12b |  |  | 25647 | -0.498 | -0.1687 | No |
| 171 | 30716 | raf1a |  |  | 25882 | -0.518 | -0.1721 | No |
| 172 | 403000 | pdpk1b |  |  | 26060 | -0.534 | -0.1737 | No |
| 173 | 571323 | foxg1c |  |  | 26100 | -0.538 | -0.1708 | No |
| 174 | 436729 | setd7 |  |  | 26299 | -0.557 | -0.1728 | No |
| 175 | 553661 | homer2 |  |  | 26345 | -0.561 | -0.1700 | No |
| 176 | 550286 | hrasa |  |  | 26472 | -0.575 | -0.1696 | No |
| 177 | 403063 | araf |  |  | 26515 | -0.580 | -0.1665 | No |
| 178 | 30637 | mdm2 |  |  | 26774 | -0.606 | -0.1701 | No |
| 179 | 406440 | prkag1 |  |  | 26779 | -0.607 | -0.1656 | No |
| 180 | 445118 | g6pca.1 |  |  | 26919 | -0.622 | -0.1653 | No |
| 181 | 114433 | igf1 |  |  | 27128 | -0.641 | -0.1670 | No |
| 182 | 321974 | prmt1 |  |  | 27316 | -0.650 | -0.1680 | No |
| 183 | 565109 | agap2 |  |  | 27527 | -0.676 | -0.1695 | No |
| 184 | 553656 | hrasb |  |  | 27554 | -0.680 | -0.1651 | No |
| 185 | 393707 | bcl6a |  |  | 27582 | -0.683 | -0.1608 | No |
| 186 | 30262 | ins |  |  | 27693 | -0.700 | -0.1590 | No |
| 187 | 553957 | il10 |  |  | 27754 | -0.710 | -0.1555 | No |
| 188 | 794088\_2 | ptena |  |  | 27793 | -0.715 | -0.1512 | No |
| 189 | 326974 | gabarapa |  |  | 27990 | -0.740 | -0.1518 | No |
| 190 | 403045\_1 | egf |  |  | 28202 | -0.773 | -0.1526 | No |
| 191 | 767751 | ccnb3 |  |  | 28375 | -0.800 | -0.1520 | No |
| 192 | 101910198 | akt1 |  |  | 28441 | -0.810 | -0.1479 | No |
| 193 | 402857 | s1pr4 |  |  | 28545 | -0.830 | -0.1448 | No |
| 194 | 30658 | rag2 |  |  | 28931 | -0.905 | -0.1501 | No |
| 195 | 100333252 | cdkn2d |  |  | 29250 | -0.990 | -0.1527 | No |
| 196 | 445289 | kras |  |  | 29269 | -0.994 | -0.1457 | No |
| 197 | 403146 | akt2l |  |  | 29342 | -1.012 | -0.1403 | No |
| 198 | 556286 | sos1 |  |  | 29488 | -1.060 | -0.1368 | No |
| 199 | 406473\_1 | pck2 |  |  | 29767 | -1.153 | -0.1369 | No |
| 200 | 394088 | homer3b |  |  | 29781 | -1.158 | -0.1285 | No |
| 201 | 563708 | skp2 |  |  | 30286 | -1.382 | -0.1339 | No |
| 202 | 100006858\_1 | csnk1e |  |  | 30290 | -1.385 | -0.1235 | No |
| 203 | 566841\_1 | crebbpa |  |  | 30571 | -1.563 | -0.1205 | No |
| 204 | 568155 | bnip4 |  |  | 30688 | -1.659 | -0.1116 | No |
| 205 | 245702\_1 | igf1rb |  |  | 31247 | -2.324 | -0.1115 | No |
| 206 | 431763\_1 | gadd45ab |  |  | 31309 | -2.449 | -0.0949 | No |
| 207 | 571431 | LOC571431 |  |  | 31422 | -2.716 | -0.0778 | No |
| 208 | 100000252\_1 | pik3r3b |  |  | 31434 | -2.733 | -0.0574 | No |
| 209 | 100000242 | zgc:92606 |  |  | 31809 | -4.711 | -0.0335 | No |
| 210 | 324140 | sgk1 |  |  | 31816 | -4.750 | 0.0023 | No |
Table: GSEA details [plain text format]

  

Fig 2: FOXO\_SIGNALING\_PATHWAY(DRE04068)      
 Blue-Pink O' Gram in the Space of the Analyzed GeneSet

  

Fig 3: FOXO\_SIGNALING\_PATHWAY(DRE04068): Random ES distribution      
 Gene set null distribution of ES for **FOXO\_SIGNALING\_PATHWAY(DRE04068)**
